# Colon cancer patients with a serious psychiatric disorder present with a more advanced cancer stage and receive less adjuvant chemotherapy - A Nationwide Danish Cohort Study

**DOI:** 10.1186/s12885-018-4879-3

**Published:** 2018-10-29

**Authors:** Linda Kaerlev, Maria Iachina, Oleg Trosko, Niels Qvist, Pernille Møller Ljungdalh, Bente Mertz Nørgård

**Affiliations:** 1 Research Unit of Clinical Epidemiology, Institute of Clinical Research, University of Southern Denmark, Kløvervænget 30, Entrance 216 ground floor east, DK- 5000 Odense C, Denmark; 2 Center for Clinical Epidemiology, Odense University Hospital, Kløvervænget 30, Entrance 216 ground floor east, DK-5000 Odense C, Denmark; 3 Department of Psychiatry (University function), The Region of Southern Denmark, Odense C, Denmark; 4 Surgical Department A, Odense University Hospital, DK-5000 Odense C, Denmark

**Keywords:** Colon and rectal cancer, Psychiatric disorders, Psychotic disorders, Mood disorders, Oncological treatment, Operation

## Abstract

**Background:**

Psychiatric patients with colorectal cancer may have delayed diagnosis and be oncologically undertreated.

**Methods:**

The Danish Colorectal Cancer Group database comprised 25,194 colorectal cancer patients (CRC), (colon cancer (CC, *n* = 16,641), rectal cancer (RC, *n* = 8553)), having an operation in 2007–2013, were alive at least 30 days after operation, of which 422 have had at least one hospital contact for a serious psychiatric disorder; ICD-10: DF20–29: primary psychotic disorders, or DF30–39: affective disorders (exposed) in a period of 3650–120 days before the operation date. Pearson chi-squared test for cancer stage was calculated. Odds Ratio (OR) with 95% confidence interval (CI) for having had a palliative vs an intended curative aim of the operative treatment for CRC patients (cohort 1), and for having an oncological treatment for each cancer site CC or RC (cohort 2 and 3) in patients with and without a psychiatric history was estimated. We adjusted the OR for: age, gender, comorbidity index, cancer stage, socio-economic position group, and educational level.

**Results:**

A higher cancer stage at the time of operation in patients with psychiatric disorders compared with patients without such a history was seen and may possibly point towards a delay in the diagnosis or in the treatment of CC in patients with psychiatric disorders. They also had decreased adjusted OR for having an oncological treatment, OR 0.55, 95% CI (0.40–0.76)), which was not explained by cancer stage. For patients with RC no difference was seen.

**Conclusions:**

Attention for CC patients with pre-existing serious psychiatric disorders is recommended.

## Background

Although cancer incidence in psychiatric patients is similar to that in the general population psychiatric disorders may have a negative impact on the prognosis of cancer [1–8]. However, psychiatric disorders may affect the advocating or acceptance of cancer diagnostic procedures or treatment as suggested for lung cancer patients [9].

For colorectal cancer (CRC) patients in Denmark, the treatment with an operation, the neoadjuvant or the adjuvant chemotherapy is carried out according to the current national guidelines. Existing significant co-morbidity may change the clinical decision-making according to the guidelines although it may compromise the oncological result [10–12].

We do not know if pre-existing psychiatric disorders in CRC patients also significantly change the CRC treatment offered to these patients.

The oncological treatment in colon cancer (CC) patients who has undergone an operation depends on the TNM-staging (tumor, node, metastasis) of the CRC and is mainly adjuvant (at pathological TNM-staging II and III), whereas stage IV treatment is considered palliative. In rectum cancer (RC) patients, neo-adjuvant radio-chemotherapy is common with the primary aim to prevent local recurrence after the operation [13–15]. A number of confounding factors such as cancer stage at diagnosis, comorbidity, age, gender, and educational level may be important for the additional treatments for CRC these patients receive, and must therefore be taken into account in analyses [8, 16–23]. We aimed to investigate if pre-existing serious psychiatric disorders affect the cancer stage at the time of operation, having palliative vs an intended curative aim of the operative treatment, or having an oncological treatment in CRC patients.

## Methods

The diagnostic process in patients with symptoms of CRC often starts with examinations and tests performed by a general practitioner, and if a suspicion of cancer is present, the person is referred to specialist evaluation and a colonoscopy at public hospitals in Denmark.

### The Danish Colorectal Cancer Group database

The study is based on the nationwide Danish Colorectal Cancer Group database (DCCG) including incident patients with CC and RC during 1 January 2007 until 31 December 2013. The DCCG does not register recurrences of the cancer after the primary operation. The database is more than 98% complete for all CRC patients in Denmark of all ages (approximately two-third CC, one-third RC) [10]. The database contains detailed data on the priority or urgency of the operation of the patients with CRC, and the variable has the categories: elective operated patients, acute operated patients, and patients with missing information regarding this issue.

The DCCG has a variable “operative aim”, with the three categories: intended curative or palliative aim of the operation, or missing information regarding this issue.

The DCCG also has a categorical variable “Treatment” based on the decisions made by the multidisciplinary cancer team (MDT). We selected for the present study only those patients in the DCCG having had an operation for CC or RC based on this variable (thus excluding those patients without having an operation, or missing information on operation (< 8% of cases), and after this restriction of the study population the variable had no missing values. The variable on treatment for all patients who were operated had the following categories: whether there was operation only, operation followed by adjuvant oncological treatments, neoadjuvant oncological treatment followed by an operation or operation with both neoadjuvant and adjuvant oncological treatments. This information was combined into a new variable: having oncological treatment (yes/no) -in addition to the operation.

The DCCG contains data on cancer type and on the clinical and the pathological cancer stage. The clinical TNM classification, cTNM or just TNM, is based on e.g. clinical, endoscopic and image diagnostic findings at the time of diagnosis and is essential in relation to decision-making on e.g. operation and evaluation of treatments. The pathological TNM classification, pTNM, is based on the histopathological examination of the operative specimen, possibly modified by per- and postoperative findings, and forms the basis for decision-making on the postoperative oncological treatment and prognostic evaluation [24]. In the DCCG, the 5th edition of The Union for International Cancer Control (UICC)'s TNM-cancer staging classification was used until 2016 (when it was replaced by the 8th edition) [25–27]. The pathological UICC cancer stage variable was grouped into stage I-II versus III-IV.

### The Civil Registration Number

Persons with a permanent address in Denmark have a unique 10-digit civil registration number, which includes information on birthday and gender, dead/alive and immigration status in The Danish Civil Registration System [28, 29]. We used this number to link the CRC cohorts in the DCCG with each CRC patient’s hospital contacts as recorded by the Danish National Patient Registry [30] for information on the exposure of serious psychiatric disorders and on comorbidity [31–33], and public registries developed by Statistics Denmark such as The Household and Family Statistics (based on The Danish Civil Registration System) as well as the personal income statistics to calculate educational level, and socio-economic position group [34].

### Inclusion criteria for the cancer cohorts

A total of 26,897 patients with an operation for CRC were retrieved from the DCCG during the 7-year period 1 January 2007 until 31 December 2013. Our study period ended just before the Danish screening programme for CRC in the general population aged 50–74 years was implemented 1 March 2014.

A priori in the study design phase we decided to make restrictions to the study population. Observations were excluded if the patients were not operated for the cancer with a registered date of the operation. To avoid that perioperative complications had influence on the choice of treatments, and to ensure that the patients were alive long enough to be offered an oncological treatment, observations were excluded if the patients died within the first 30 days after their operation.

For the analyses of CRC patients having received a palliative vs an intended curative aim of the operative treatment all the CRC patients were included for the analyses (cohort 1) and split by having a pre-existing psychiatric disorder (yes/no).

For the analyses of CRC patients having received at least one oncological treatment in the DCCG before or after the cancer operation we stratified the analyses into CC patients only (cohort 2) and RC patients only (cohort 3).

### Pre-existing serious psychiatric disorders according to The National Patient Registry

Each CRC patient was linked to The National Patient Registry to collect information on hospital contact for: ICD-10 DF20–29: Schizophrenia, schizotypal and delusional disorders (primary psychotic disorders), or ICD-10 DF30–39 mood (affective) disorders as the primary diagnosis in the time-period from 10 years to 120 days prior to the date of the CRC operation [30]. If yes, the patients were regarded as exposed, otherwise not-exposed. Exclusion of psychiatric disorders diagnosed up to 120 days before the date of the cancer operation was done to avoid that natural psychological crisis reactions as a result of the cancer diagnosis were misclassified as pre-existing psychiatric disorders.

### Measures of the outcomes

The CRC patients (with and without a psychiatric history) were examined in the DCCG for the cancer stage at the time of the operation, and for having received palliative vs an intended curative aim of the operative treatment, and for receiving at least one oncological treatment either before or after the cancer operation [10].

### Demographic characteristics, cancer stage and possible confounders

Using the unique civil registration number, we obtained the gender and date of birth, and migration and death. The UICC pathological cancer stage variable was taken from the DCCG. Information on co-morbidity was obtained from The National Patient Registry merged with the DCCG, with calculation of a slight modification of the Charlson Co-morbidity Index by excluding all contacts with CRC prior to the date of the CRC operation from the comorbidity index score [31–33]. Each of the 19 categories of comorbid diseases was assigned a severity score between 0 and 6, and the comorbidity index was calculated as the sum of the scores (possible range between 0 and 37 for each patient). The predictive value of the comorbidity index is high [32]. Cancer diagnoses within 150 days before the date of CRC operation may be CRC misclassified as other cancers and was not included in the comorbidity index. The comorbidity index was re-coded as a score into a categorical variable (0, 1, and above 1 point).

The registries developed by Statistics Denmark was used to calculate socio-economic position, which was included as quartiles in the analyses (for the main analyses yearly income below DKK 143,927, DKK from 143,927–178,573, DKK from 178,573-247,677.4, and above DKK 247,677.4) [34]. Educational level from Statistic Denmark was included in the analyses as a categorical variable: 1) elementary school only, 2) upper secondary education/vocational education and training 3) qualifying educational programmes/vocational bachelor education/bachelor programmes/masters programmes/PhD programmes.

### Analysis

We compared the CRC patients, who have had a psychiatric disorder with the CRC patients without a psychiatric history with respect to demographic characteristics and cancer stage at the time of the operation with Pearson chi-squared tests. Continuous variables were compared between the groups with Students t-test. The date of the operation was the index date in the study.

Specifically for the descriptive analyses for the patients with CRC and the analyses for cancer stage, we used all the patients with CRC and we further stratified the analyses into the acute operated CRC patients only, and CRC patients who had undergone an elective operation only, and excluding those patients with missing information regarding this issue.

The distribution of the cancer treatment by pre-existing psychiatric disorder status was calculated (provided in Table 3). For the analyses of palliative vs an intended curative aim of the operative treatment, we used all the CRC patients. For the analyses of the oncological treatment outcomes all the CRC patients was split by anatomical site of the cancer, CC or RC. We estimated the odds ratio (OR) with 95% confidence intervals (CIs) for the patients with a psychiatric history for having received palliative vs an intended curative aim of the operative treatment, and for having at least one oncological treatment (before or after the operation) by logistic regression.

We adjust for each of the possible important confounders as described in the CRC literature: gender, age and the Charlson comorbidity index score, pathological cancer stage (UICC cancer stage I-II versus III -IV) [24], socio-economic position (four levels), and educational level (three levels) in the adjusted analysis model. After each adjustment step we evaluated the corresponding OR with 95% CI.

We have analyzed the correlation between each of the possible confounders and between having had a psychiatric disorder and the confounding factors.

Finally, sub-analyses were performed with restriction to primary psychotic disorders only, affective disorders only, and to elective operated patients or to acute operated patients only.

## Results

**Fig. 1:**
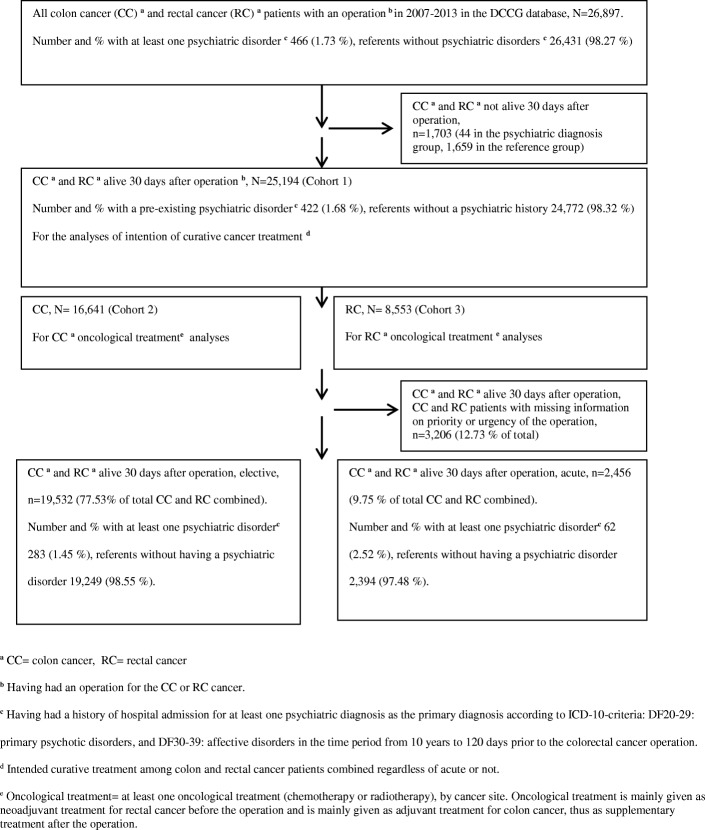
Selection of colon and rectal cancer patients from the DCCG for the study

Figure 1 shows the selection of all the CRC (cohort 1), CC and RC only (cohort 2 and 3)) used for the present study, and the subgroups of elective and the acutely operated CRC used for the supplementary descriptive statistic in Table 1. A total of 1703 CRC patients (44 in the psychiatric diagnosis group, 1659 in the reference group) died within the first 30 days after operation and were excluded, leaving a total of 25,194 CRC patients (422 with a pre-existing psychiatric history, and 24,772 in the reference group) alive at least 30 days after their operation for the study (Fig. 1). The number of CRC patients increased from 2007 to 2013: in 2007 3147, in 2008 3577, in 2009 3517, in 2010 3596, in 2011 3688, in 2012 3803, to 3866 in 2013. The 422 CRC patients with at least one serious psychiatric diagnosis in The National Patient Registry (301 patients with CC, and 121 patients with RC) had altogether 572 psychiatric diagnoses with the majority of the diagnoses within DF30–39: affective disorders (445 (77.8%)), and less than one-fourth 127 (22.2%) was within DF20–29: primary psychotic disorders.

**Table 1:** Descriptive table. Comparison of all colorectal cancer (CRC) patients alive 30 days after the operation stratified by pre-existing psychiatric history. Similarly with restriction to elective patients only

	All CRC patients (cohort 1, *N* = 25,194) ^a^					Elective CRC patients only (*N* = 19,532 (after exclusion of missing) ^b^					Acute CRC patients only (*N* = 2456) (after exclusion of missing)^b^				
	Psychiatric disorder ^c^ cases (*N* = 422)		Referents without psychiatric disorders ^c^ (N = 24,772)		*p*-value, ^d^	Psychiatric disorder ^c^ cases (*N* = 283)		Referents without psychiatric disorders ^c^ (N = 19,249)		*p*-value, ^d^	Psychiatric disorder ^c^ cases (*N* = 62)		Referents without psychiatric disorders ^c^ (*N* = 2394)		*p*-value, ^d^
	N	%	N	%		N	%	N	%		N	%	N	%	
Cancer stage															
Low, UICC stage (I, II)	163	38.63	11,058	44.64		148	52.30	10,276	53.38		12	19.35	667	27.86	
Advanced stage (III,IV)	227	53.79	12,199	49.25	0.038	122	43.11	8261	42.92	0.72	48	77.42	1626	67.92	0.283
Missing information	32	7.58	1515	6.12		13	4.59	712	3.70		<=3 ^g^	2.93	101	4.22	
	Mean	SD ^e^	Mean	SD ^e^		Mean	SD ^e^	Mean	SD ^e^		Mean	SD ^e^	Mean	SD ^e^	
Age (years)	68.08	12.33	70.42	11.31	0.001	66.69	11.55	69.87	11.08	0.001	69.82	13.71	71.31	12.11	0.171
Gender															
Men	171	40.52	13,226	53.55		119	42.05	10,366	53.85		19	30.65	1166	48.71	
Women	251	59.48	11,506	46.45	0.001	164	57.95	8883	46.15	0.001	43	69.35	1228	51.29	0.005
Socio-economic position, n (%) ^f^															
Quartile 1	119	28.20	5857	23.64		74	26.15	4524	23.50		23	37.10	632	26.40	
Quartile 2	130	30.81	5847	23.60		90	31.80	4619	24.00		19	30.65	592	24.73	
Quartile 3	96	22.75	5882	23.74		71	25.09	4769	24.78		14	22.58	548	22.89	
Quartile 4	46	10.90	5930	23.94	0.001	39	13.78	4935	25.64	0.001	4	6.45	493	20.59	0.041
Missing information	31	7.35	1256	5.07		9	3.18	402	2.09		–	–	–	–	
Education															
Primary education/elementary school	180	42.65	9549	38.55		116	40.99	7540	39.17		31	50.00	995	41.56	
Short education /professional training	141	33.41	9689	39.11		101	35.69	7847	40.77		22	35.48	887	37.05	
Middle long education or university	66	15.64	3695	14.92	0.12	54	19.08	3018	15.68	0.25	6	9.68	326	13.62	0.486
Missing information	35	8.29	1839	7.42		12	4.24	844	4.38		3	4.84	186	7.77	
Charlson co-morbidity index score at date of the cancer operation, number (%)															
0	245	58.06	16,912	68.27		168	59.36	13,326	69.23		34	54.84	1646	68.76	
1	82	19.43	3537	14.28		54	19.08	2740	14.23		12	19.35	321	13.41	
> 1	95	22.51	4323	17.45	0.001	61	21.55	3183	16.54	0.002	16	25.81	427	17.84	0.067

^a^All CRC patients (cohort 1) with an operation in Denmark 2007–2013 according to The Danish Colorectal Cancer Group database, who were alive at least 30 days after operation, by pre-existing psychiatric history

^b^Elective (*n* = 19,532) and acute (*n* = 2456) operated CRC patients. Both populations split by a pre-existing psychiatric history. Missing information regarding acute or elective priority or urgency of the operation (*n* = 3206, 12.73% of total CRC)

^c^Psychiatric disorder cases = having had a history of hospital admission for at least one psychiatric diagnosis as the primary diagnosis according to ICD-10-criteria: DF20–29: primary psychotic disorders, and DF30–39: affective disorders in the time period from 10 years to 120 days prior to the colorectal cancer operation

^d^Pearson Chi-square test or t-test, *p*-value

^e^SD = standard deviation,

^f^The socio-economic position was defined by level of yearly income according to the definition used by Statistics Denmark, quartiles (for the main analyses below DKK 143,927, DKK from 143,927–178,573, DKK from 178,573-247,677.4, and above DKK 247,677.4)

^g^Anonymised number (number = 1 or 2) according to the Danish data protection recommendation, (percentage)

For information on priority or urgency of the operation, the 25,194 patients with CRC was registered as electively operated patients only (*n* = 19,532, 77.53%), as acutely operated CRC patients only (*n* = 2456, 9.75% of total CRC), whereas the rest (*n* = 3206, 12.73% of total CRC) was registered with missing information regarding this issue (Table 1).

For information on “operative aim”, the majority of the CRC patients was registered with having had an intended curative operation (n = 19,700, 78.19%) and far fewer had a palliative aim of the operation (*n* = 2310, 9.17%), and missing values (*n* = 3184, 12.64%).

Characteristics of the overall group of CRC patients, (cohort 1) as well as for elective patients only or acutely operated patients only are given in Table 1.

Information on cancer stage was available for 23,647 patients (*n* = 390; 92.42% with, and n = 23,257; 93.88% without a preexisting psychiatric disorder) and missing for 1547 patients (*n* = 32; 7.58% with and *n* = 1515; 6.12% without a preexisting psychiatric disorder).

The CRC patients with a psychiatric disorder differed statistically significantly from the patients without a psychiatric disorder at the date of the cancer operation by more women, lower mean age and socio-economic position group, higher comorbidity index scale, and cancer stage at the time of the operation (high cancer stage 53.79 versus 49.25%, *p* = 0.038), but not by educational level. Slightly more CRC patients with a psychiatric disorder were treated acutely (62/422*100 = 14.7%) compared with CRC patients without a psychiatric history (2394/24772*100 = 9.7%) (data not shown).

The acute CRC patients in general had a higher cancer stage than their non-acute registered counterparts (68%/ 49%). In a sub-analysis on electively operated CRC patients only, similar findings were seen except that the cancer stage no longer differed between patients with and without a psychiatric history.

Table 2 shows descriptive statistics for the overall group of CRC patients split by cancer site: CC (cohort 2) and RC (cohort 3). As for CRC patients in cohort 1, similar findings were seen for the subgroup of CC patients including a higher cancer stage (54.49% versus 51.03%, *p* = 0.03) at the time of the CC operation. For RC, the patients with a psychiatric history only differed from those without by lower mean age, lower socio-economic position group, and more comorbidity.

**Table 2:** Descriptive table. Comparison of all colorectal cancer patients alive 30 days after the operation stratified by cancer site (colon versus rectum) and by pre-existing psychiatric history

	All colon cancer patients ^a^					All rectal cancer patients ^b^				
	Psychiatric disorder ^c^ cases (*N* = 301)		Referents without psychiatric disorders ^c^ (*N* = 16,340)		*p*-value, ^d^	Psychiatric disorder^c^ cases (*N* = 121)		Referents without psychiatric disorders^c^ (*N* = 8, 432)		*p*-value,^d^
	N	%	N	%		N	%	N	%	
Cancer stage ^b^										
Low, UICC stage (I, II)	115	38.21	7220	44.19		48	39.67	3838	45.52	
Advanced UICC stage (III,IV)	164	54.49	8339	51.03	0.03	63	52.07	3860	45.78	0.377
Missing information	22	7.31	781	4.78		10	8.26	734	8.70	
	Mean	SD ^e^	Mean	SD ^e^		Mean	SD ^e^	Mean	SD ^e^	
Age (years)	68.44	12.46	71.13	11.25	0.001	67.22	12.02	69.03	11.29	0.041
Gender										
Men	107	35.55	8109	49.63		64	52.89	5157	61.16	
Women	194	64.45	8231	50.37	0.001	57	47.11	3275	38.84	0.064
Socio-economic position, n (%) ^f^										
Quartile 1	81	26.91	3907	23.91		38	31.40	1950	23.13	
Quartile 2	99	32.89	3926	24.03		31	25.62	1921	22.78	
Quartile 3	67	22.26	3812	23.33		29	23.97	2070	24.55	
Quartile 4	31	10.30	3841	23.51	0.001	15	12.40	2089	24.77	0.016
Missing information	23	7.64	854	5.23		8	6.61	402	4.77	
Education										
Primary education/elementary school	127	42.19	6322	38.69		53	43.80	3227	38.27	
Short education / professional training	98	32.56	6266	38.35		43	35.54	3423	40.60	
Middle long education or university	47	15.61	2475	15.15	0.19	19	15.70	1220	14.47	0.502
Missing information	29	9.63	1.277	7.82		6	4.96	562	6.67	
Charlson co-morbidity index score at date of the cancer operation, number (%)										
0	173	57.48	10,819	66.21		72	59.50	6093	72.26	
1	56	18.60	2428	14.86		26	21.49	1109	13.15	
> 1	72	23.92	3093	18.93	0.007	23	19.01	1230	14.59	0.005

^a^All colon cancer patients (cohort 2) with an operation in Denmark 2007–2013 according to The Danish Colorectal Cancer Group database, who were alive at least 30 days after operation, by a pre-existing psychiatric history

^b^All rectum cancer patients (cohort 3) with an operation in Denmark 2007–2013 according to The Danish Colorectal Cancer Group database, who were alive at least 30 days after operation, by a pre-existing psychiatric history

^c^Psychiatric disorder cases = having had a history of hospital admission for at least one psychiatric diagnosis as the primary diagnosis according to ICD-10-criteria: DF20–29: primary psychotic disorders, and DF30–39: affective disorders in the time period from 10 years to 120 days prior to the colorectal cancer operation

^d^Pearson Chi-square test or t-test, p-value

In Table 3, the crude and adjusted OR for having undergone at least one oncological treatment for the CRC, CC, or RC patients with a psychiatric history compared with those without psychiatric disorders is shown. The OR adjusted for age, gender, comorbidity index score, cancer stage, socio-economic position, and educational level is provided in Table 3. For CRC patients with a psychiatric history compared to those without, no statistical significant differences were seen between these two groups for having received a palliative vs an intended curative aim of the operative treatment.

**Table 3:** The distribution and odds ratio for palliative vs an intended curative aim of the operation among operated colorectal cancer (CRC) patients, and for oncological treatment for colon and rectum cancer by pre-existing psychiatric history

Study population, (N) -number with information available	Cancer procedure	Patients with psychiatric disorder^a^ n (%)	Patients without psychiatric disorders^a^ n (%)	Crude OR (95% CI), (reference no psychiatric disorder)	Adjusted OR ^b^ (95% CI) (reference no psychiatric disorder)
Cohort 1 All CRC (22,010 with information)					
Aim of the operative treatment^c^					
Curative		308 (72.99)	19,392 (78.28)		
Palliative		38 (9.00)	2272 (9.17)	1.05 (0.75–1.48)	0.93 (0.62–1.39)
Missing		76 (18.01)	3108 (12.60)		
Cohort 2 Colon cancer (16,641)					
Oncological treatment^d^					
No		193 (64.12)	9519 (58.26)		
Yes		108 (35.88)	6821 (41.74)	0.78 (0.62–0.99)	0.55 (0.40–0.76)
Cohort 3 Rectum cancer (8553)					
Oncological treatment^d^					
No		64 (52.89)	4011 (47.57)		
Yes		57 (47.11)	4421 (52.43)	0.81 (0.56–1.16)	0.72 (0.46–1.11)

^a^Psychiatric disorder cases = having had a history of hospital admission for at least one psychiatric diagnosis as the primary diagnosis according to ICD-10-criteria: DF20–29: primary psychotic disorders, and DF30–39: affective disorders in the time period from 10 years to 120 days prior to the colorectal cancer operation

^b^Odds ratio (OR) with 95% confidence interval (CI), adjusted for age, gender, Charlson Comorbidity Index score, cancer stage at the time of operation, educational level, socio-economic position

^c^Curative or intended palliative aim of the operative treatment among colon and rectal cancer patients combined regardless of acute or not

^d^Oncological treatment = Received at least one oncological treatment (chemotherapy or radiotherapy), by cancer site

Similar analysis for acute operated patients only, also showed no significant differences in the percentage of CRC patients receiving palliative treatment between the patients with and without a psychiatric history *p* = 0.797 (data not shown).

For CC patients with a psychiatric history compared to CC patients without a psychiatric history the adjusted OR for at least one oncological treatment was OR 0.55, 95% CI (0.40–0.76). The association was present both before and after adjustment for cancer stage. For the RC group with a psychiatric history no differences were seen for having received at least one oncological treatment, OR = 0.72, 95% CI (0.46–1.11).

In sub-analyses with restriction to primary psychotic disorders, or to affective disorders only, the results were still statistically significantly decreased for oncological treatment of CC. For each of the outcomes we investigated, whether stepwise adjustment for any of the possible confounding factors: age, gender, Charlson Comorbidity Index score, the cancer stage at the time of operation, socio-economic position, and educational level had statistically significantly influenced the OR of psychiatric history. This was not the case. Removing educational level as a confounder in the model and only adjusting for five confounding factors did not change the results. Furthermore, for each of the investigated outcomes, we calculated the correlation between the estimated coefficients and we found that the highest correlation between the estimated confounders and psychiatric history was approximately 0.05.

## Discussion

A higher cancer stage at the time of the operation for CC patients but not statistically significantly for RC patients may most likely points to a delay in the diagnosis or in the treatment of CC patients with a pre-existing serious psychiatric diagnosis, but the reason is unknown and may be patient related, or related to the primary healthcare system or hospital-related factors [35–37].

CC patients with a psychiatric history had a reduced OR for having received at least one oncological treatment (often as an adjuvant treatment) and oncological under-treatment of CC patients with a psychiatric history is possible. The association was present both before and after adjustment for cancer stage, and was also seen for each of the two serious psychiatric disorder groups. In the current Danish and European guidelines for CRC, psychiatric disorders are not considered a contraindication for oncological treatment. It is mentioned that comorbidity in general can change clinical decision-making, especially in older CRC patients [10–12]. CRC patients with a psychiatric history are, however, in average younger than CRC patients without a psychiatric history. In RC patients, the OR of having had at least one oncological treatment was also decreased but however not statistically significantly affected by a history of psychiatric disorder, where oncological treatment often is given neoadjuvant to the RC operation.

This is to the best of our knowledge the first study to report differences in oncological treatment due to pre-existing primary psychotic or affective disorders in CC patients having an operation, and with the possibility to adjust for age, gender, comorbidity, and cancer stage. A study on all patients with a first cancer diagnosis of all types in 1990–2013 in the Finnish Cancer Registry, showed excess mortality in people with a history of psychotic and substance use disorders, whereas adjusting for cancer treatment decreased the differences [4]. A study from Australia on all cancers patients combined and psychiatric disorders from 1988 to 2007 as exposure showed that the psychiatric patients, especially those with psychoses and depression, received significantly fewer CRC operations and had higher all-cause mortality than the general population of CRC patients [1]. The study did not look at oncological treatment for CRC according to the current guidelines. The reasons and the clinical consequences needed to be investigated further. Possible explanations for further studies include delays in symptom recognition or in the initial presentation of the CC, difficulties in communication, diagnostic delays or other factors in the primary or secondary healthcare system.

### Strengths and limitations

The strengths of our study were the large Nationwide Danish study population with close to complete background information and follow-up from highly valid registries with prospectively collected patient data thus minimizing selection bias, comprehensive measures of potential confounders and nearly complete information on the chosen diagnoses and treatments for the entire study population [10, 30].

There may have been a difference in the initial CRC stage and comorbid disease stages in patients with a pre-existing psychiatric disorder compared with patients without a pre-existing psychiatric condition, and especially among those patients who died within the first 30 days. By only selecting patients, alive at least 30 days after surgery for the study population may have removed proportionally more patients with a pre-existing psychiatric disorder with a more advanced disease. The finding of a more advanced disease in the psychiatric group might thus be underestimated, although we have adjusted the analyses for cancer stage as a possible confounding factor.

The “treatment” variable had less than 8% missing values. Still, it is possible with underreporting in this variable of adjuvant oncological treatment. Since this rather few missing values of the treatment variable is not expected to differ between patients with and without having pre-existing psychiatric disorder, it is not expected to bias the results [23].

We have included possible confounders previously mentioned in the CRC literature in the models. Educational level may to some degree be correlated with socio-economic position. We checked for each of the investigated outcomes whether stepwise adjustment for the possible confounding factors age, gender, Charlson Comorbidity Index score, cancer stage at the time of operation, socio-economic position, and educational level had statistically significantly influenced the OR. This was not the case. Furthermore, the coefficients estimates of the possible confounding factor socio-economic position and educational level was only weakly correlated (correlation coefficient approximately 0.05) to the estimate of psychiatric history. Therefore such correlations may not explain the findings. Hospital treatments are free of charge in Denmark and the majority of patients with symptoms of a severe psychiatric disorder will be referred at least once to and diagnoses at a public hospital and thus registered in The National Patient Registry. However, in Denmark and e.g. Finland a reduction in psychiatric hospital beds and an increase in out-patient services have occurred in the recent decades [4] and our study does not cover patients with e.g. depressive disorders only seen by general practitioners, which may lead to an underestimation of the associations seen.

Since the psychiatric medication may constitute a contraindication for oncological treatment for CC, and oncological treatment requires patient compliance which can be difficult for patients with psychiatric disorders to follow, these factors may to some degree explain the findings [10–12].

Other possible explanations may include the health behaviors of the patient, difficulties with regard to communication, or failures in the primary or secondary healthcare system for various reasons.

## Conclusions

A higher cancer stage at the time of operation point towards a possible delay in the diagnosis or in the treatment of CC in patients with a serious psychiatric disorder and these patients also had a decreased OR for receiving at least one oncological treatment. The reasons for these differences need to be investigated further e.g. in the underlying patient records. Attention for CC patients with serious psychiatric disorders is recommended.

## Abbreviations

CC: Colon cancer patients
OR: Odds Ratio with CI: 95% confidence interval
CRC: Colorectal cancer patients
DCCG: The Danish Colorectal Cancer Group database
MDT: Multidisciplinary cancer team
RC: Rectal cancer patients
TNM-staging: Tumor, node, metastasis
UICC: The Union for International Cancer Control
